# Measures of social connectedness in adult populations: a systematic review

**DOI:** 10.1186/s12889-024-20779-0

**Published:** 2024-12-05

**Authors:** Ruth Plackett, Joe Hulin, Clara Mukuria, Mark Clowes, Sheena E. Ramsay, Liam Spencer, Emma A. Adams, Jennifer Dykxhoorn, Kate Walters, David P. J. Osborn, Victoria Zamperoni, Oliver Jones, Scott Weich

**Affiliations:** 1 UCL Research Department of Primary Care & Population Health, Royal Free Hospital, University College London, Upper 3rd Floor, Rowland Hill Street, London, NW3 2PF UK; 2 University of Sheffield, Sheffield, UK; 3 Newcastle University, Newcastle upon Tyne, UK; 4 University College London, London, UK; 5 Camden and Islington NHS Foundation Trust, London, UK; 6 Mental Health Foundation, London, UK; 7 McPin Foundation, London, UK

**Keywords:** Review, Social connectedness, Public health, Loneliness, Social support, Psychometric properties

## Abstract

**Background:**

Poor social connectedness has been identified as a risk factor for poor mental health but there is a lack of standardisation in how it is measured. This systematic review aimed to identify suitable measures of social connectedness for use in UK adult general populations.

**Methods:**

Searches were undertaken in two stages to identify: (1) measures of social connectedness from review articles and grey literature and (2) studies reporting on the psychometric properties of the identified measures. Grey literature and five databases were searched: MEDLINE, Embase and PsycINFO; CINAHL and Web of Science. Studies based on UK adult general populations (16–65 years) or other English language speaking countries with similar cultures (US, Canada, Ireland, Australia and New Zealand) were included. Psychometric evidence was extracted relating to six general domains: conceptual model, content validity, reliability, construct validity, scoring and interpretability, and respondent burden and presentation. A narrative synthesis summarised these psychometric properties.

**Results:**

Stage (1) 2,396 studies were retrieved and, 24 possible measures of social connectedness were identified; stage (2) 6,218 studies were identified reporting on psychometrics of identified measures and 22 studies were included. These studies provided psychometric evidence for 10 measures, and we did not find psychometric studies for the other identified measures. Six measures (6/10, 60%) reported assessing loneliness and four (4/10, 40%) reported assessing social support but there was a degree of overlap between the assessments of each concept. There was good evidence of reliability across measures, 90% (9/10) had adequate internal consistency, but evidence of content validity was only available for one scale. Five measures (5/10, 50%) reported on at least half of the psychometric criteria, and these were: UCLA-3 (for loneliness), and MSPSS, F-SozU K-6, SPS-10 and SPS-5 (for social support).

**Conclusions:**

This review identified ten social connectedness measures, and identified UCLA-3, MSPSS, F-SozUK-6, SPS-10, and SPS-5 as having the most robust psychometric properties for the UK adult population. Further testing is required to establish content validity, and to clarify the definition and conceptualisation of social connectedness, to enable standardisation in the approach to measuring social connectedness.

**Supplementary Information:**

The online version contains supplementary material available at 10.1186/s12889-024-20779-0.

## Background

Social connectedness has been described as a person’s subjective experience of belonging and relatedness to others [1]. This definition extends beyond the objective assessment of an individual’s social world, such as social network size or frequency of contacts with others [2]. Social connectedness can be seen to encompass the perceived quality or adequacy of social support available to an individual and feelings of loneliness or isolation resulting from absence of close relationships or integration in a social network [3]. Poor social connectedness has been identified as a key risk factor for poor health, with studies showing that loneliness and lack of adequate social support are both associated with increased mortality [4, 5], and higher rates of depression and anxiety [5–7].

Given the significant impact of social connectedness on health outcomes and the costs associated with that, improving social connectedness has been identified as a public health priority in England [8], with government strategy highlighting the need for a more connected society [9]. One objective of this strategy is to develop a better understanding of how loneliness can be measured consistently to understand who is at increased risk and evaluate interventions for loneliness or social connectedness [9]. This led to the development of the Office of National Statistics (ONS) recommended package of measures to assess loneliness, for use in the Public Health Outcomes Framework [10]. However, further work is needed to identity and assess the validity and reliability of measures which assess wider aspects of social connectedness (e.g., perceived social support and sense of belonging) for use in evaluative public mental health research.

This review is part of a wider programme of work by the National Institute for Health and Care Research, School for Public Health Research (SPHR), seeking to develop a core public mental health outcome set [11]. Core outcome sets are recommendations for what should be measured and reported for research in a specific area. Three stakeholder workshops conducted by SPHR researchers and voluntary sector partners were undertaken in London in September 2019 (*n* = 38) with members of the public, public mental health practitioners, commissioners, and researchers, to establish which domains of public mental health to focus on. The workshops identified social connectedness as one of the important domains. Developing a comprehensive core outcome set of social connectedness measures is important to help researchers, practitioners, and policymakers to employ a more consistent and robust approach to measuring social connectedness. This in turn will make findings across evaluations more readily comparable (hence easier to interpret), which will help to inform policy and practice [12, 13]. Additionally, a more consistent approach to measuring social connectedness would aid the interpretation of longitudinal observational research to estimate the effect of social connectedness on the health and wellbeing of specific community groups and the general population [12].

To develop a core outcome set for social connectedness it is important to collate and understand the evidence for the psychometric properties of these measures, specifically the reliability and validity of these measures. Understanding these aspects of a measure helps to inform researchers and practitioners of the most appropriate measure to use for research and clinical practice [14, 15]. Previous research has begun to collate evidence of the psychometric properties of mental health measurement scales more broadly and for those with mental disorders but has not yet included measures of social connectedness in the adult general population [13, 16, 17]. This review will focus on the measurement of two key aspects of the concept of social connectedness that stakeholders in the wider SPHR programme identified as important to understanding social connectedness: subjective feelings of loneliness and perceived adequacy of social support. This systematic review aims to identify and describe measures of social connectedness used in the study of mental health outcomes, suitable for use in UK adult general populations, and to synthesise evidence of their psychometric properties.

## Methods

### Registration

This systematic review protocol was registered in the PROSPERO database (Registration no: CRD42020186218) and outlines the background, aims and procedures for several reviews that were conducted for different public health outcomes that were identified in previous workshops as public health priorities [18]. We performed this review following the Preferred Reporting Items for Systematic Reviews and Meta-Analyses (PRISMA) checklist [19].

### Search strategy

The review process was carried out in two stages. First stage searches were developed to identify measures of social connectedness suitable for use in public mental health research using existing review articles. Second stage searches were then undertaken to identify studies reporting the psychometric properties of measures identified in first stage searches.

#### Stage one: identification of measures

A comprehensive search of several databases, including MEDLINE, Embase and PsycINFO; CINAHL and Web of Science, was conducted from January 2000-June 2020. Terms for social connectedness were combined with terms for population health (e.g. “public health”), instruments (such as index, tool or proprietary names) and a comprehensive search filter for outcome terms [20]. As initial searches retrieved a very large number of potentially relevant articles with poor specificity, the McMaster University “best balance” of sensitivity/specificity was used to limit the search results to review articles (see Additional file 1 for the search strategy for each database) [21]. Key mental health websites were also searched to identify possible relevant materials.

#### Stage two: appraisal of psychometric properties

Searches were conducted to identify studies that reported information on the psychometric properties on the measures identified in stage one, to determine the reliability and validity of the included measures in the UK adult general population. The following databases were searched for all studies up until January 2021: MEDLINE, Embase and PsycINFO; CINAHL and Web of Science. Web searches and hand searches of reference lists of the included studies were also undertaken to identify original scale development papers and user manuals of the included measures. The Terwee filter [22] was used to search for studies evaluating the psychometric properties of measures identified for social connectedness. This filter was designed for MEDLINE but selected terms drawn from it were used for the other databases (see Additional file 1 for the search strategy for each database).

### Inclusion/exclusion criteria

#### Stage one: identification of measures

For stage one, studies were eligible for inclusion if they were published review articles (literature reviews, systematic, narrative, or meta-analysis), published in English since 2000, focused on general adult populations (16 years or older) and measures of social connectedness. We also included grey literature (reports, guides, and briefing documents) reporting on measures of public mental health in the general adult population. We excluded reviews that were solely focused on specific sub-populations including children and young people, older adults (ages 65 or older), clinical populations, students, prison populations, war veterans, participants from work-place settings or employee groups.

We used the Haslam and colleagues definition of social connectedness: “The sense of belonging and subjective psychological bond that people feel in relation to individuals and groups of others” [2]. Therefore, we restricted measures of social connectedness to those that assess subjective views, perceptions, or experiences of social connectedness. We excluded single item measures, as social connectedness is a relatively broad and complex construct, which cannot be adequately captured by a single item. Measures designed specifically for certain populations (e.g., specific conditions, hospital, or occupational settings) were also excluded. Since we only wanted to include measures suitable for use in public health research, we also excluded those that required special qualifications or specialist training to administer the measure.

#### Stage two: appraisal of psychometric properties

In stage two, only published, peer-reviewed, English language research studies were included. Studies that included samples from adult general populations based in the UK or other English language speaking countries with similar cultures were included. As above, we excluded studies focused on specific sub populations (children and young people, older adults (ages 65 or older), clinical populations, students, prison populations, war veterans, participants from work-place settings or employee groups. Information on psychometric properties was not consistently reported in peer reviewed publications so, we also included original development papers, and user guides/online guidance, to evaluate these domains.

### Study selection

Three researchers (JH, LS, VZ) screened the titles and abstracts of papers identified in both stages of searching against the inclusion criteria using Rayyan. Two researchers (JH and LS) independently double screened 20% of records at this stage to minimise systematic and random errors. Studies that met the inclusion criteria and studies that did not provide sufficient information in the title and abstract were selected for full-text screening. Two researchers (JH and VZ) independently assessed the full text studies for their eligibility to be included in the review. Discrepancies in eligibility of papers were resolved through discussion with the wider study team.

### Data extraction and synthesis

Data were extracted from selected texts using data extraction forms developed in Microsoft Excel for each stage of the review and piloted on a small number of included papers by four researchers (JH, VZ, LS, CM). Overall, 20% of data were independently extracted by at least two researchers (JH, VZ, LS, CM) and discrepancies were resolved through discussion with the wider study team. For stage one, we collected data on population, setting, review type and outcome measures. For stage two, we collected data on the outcome measures, sample characteristics and the psychometric properties of measures. A simplified 18-item checklist [23] was used to evaluate the psychometric properties of included measures across six general domains: conceptual model, content validity, reliability, construct validity, scoring and interpretability, and respondent burden and presentation. This checklist was chosen as it comprehensively assesses psychometric properties using a simplified and user-friendly checklist that has been shown to have good agreement between those with differing levels of experience with measurement theory [23]. Table 1 provides a definition and examples of evidence for each domain. We assessed each study, noting if each domain of psychometric evidence was present or absent. Characteristics of identified measures and studies were narratively synthesised.

**Table 1:** Description of the six psychometric properties extracted from included studies using an 18-item checklist

Domain	No. of items in checklist	Definition	Examples of evidence
Conceptual model	3	The rationale and description of the concepts and populations it is intended to assess	Definition of the concept and the intended population and whether the scale intends to measure a single construct or multiple subscales.
Content validity	3	The comprehensiveness and relevance of the included items	Members of the intended respondent population and content experts are included in the development of the measure. The methods used to develop the items included in the scale are reported e.g., focus groups with experts.
Reliability	2	Consistency of which the scale measures the intended construct	Adequate internal consistency across the included items and consistency of measurement over time (i.e. test-retest reliability).
Construct validity	4	The degree to which a scale measures the intended theoretical construct	Quantitative justification that single or multiple subscales exists using factor analysis or response theory. Evidence of responsiveness to change - both test-retest reliability and the detection of expected changes in adult general populations. The degree to which the measure correlates with other scales that measure similar constructs or other clinical indicators. Evidence that the measure differentiates between groups known to differ on the variable of interest.
Scoring and interpretation	3	The degree to which the meaning of scores is easily understood	Clearly describe the scoring system. Details of how scores should be computed, including details for how to manage missing responses. Detailed guidance on how to interpret scores is also available e.g., to calculate cut-off scores.
Respondent burden and presentation	3	The demands placed on the respondent or those administering the measure	Time taken to complete the questionnaire. For this review any measures with 20 items or fewer were deemed to be appropriate for public mental health research even if time was not reported. Literacy level of a reading age of 11–12 years is reported. The full scale is publicly available.

## Results

### Study identification

#### Stage one

**Fig. 1:**
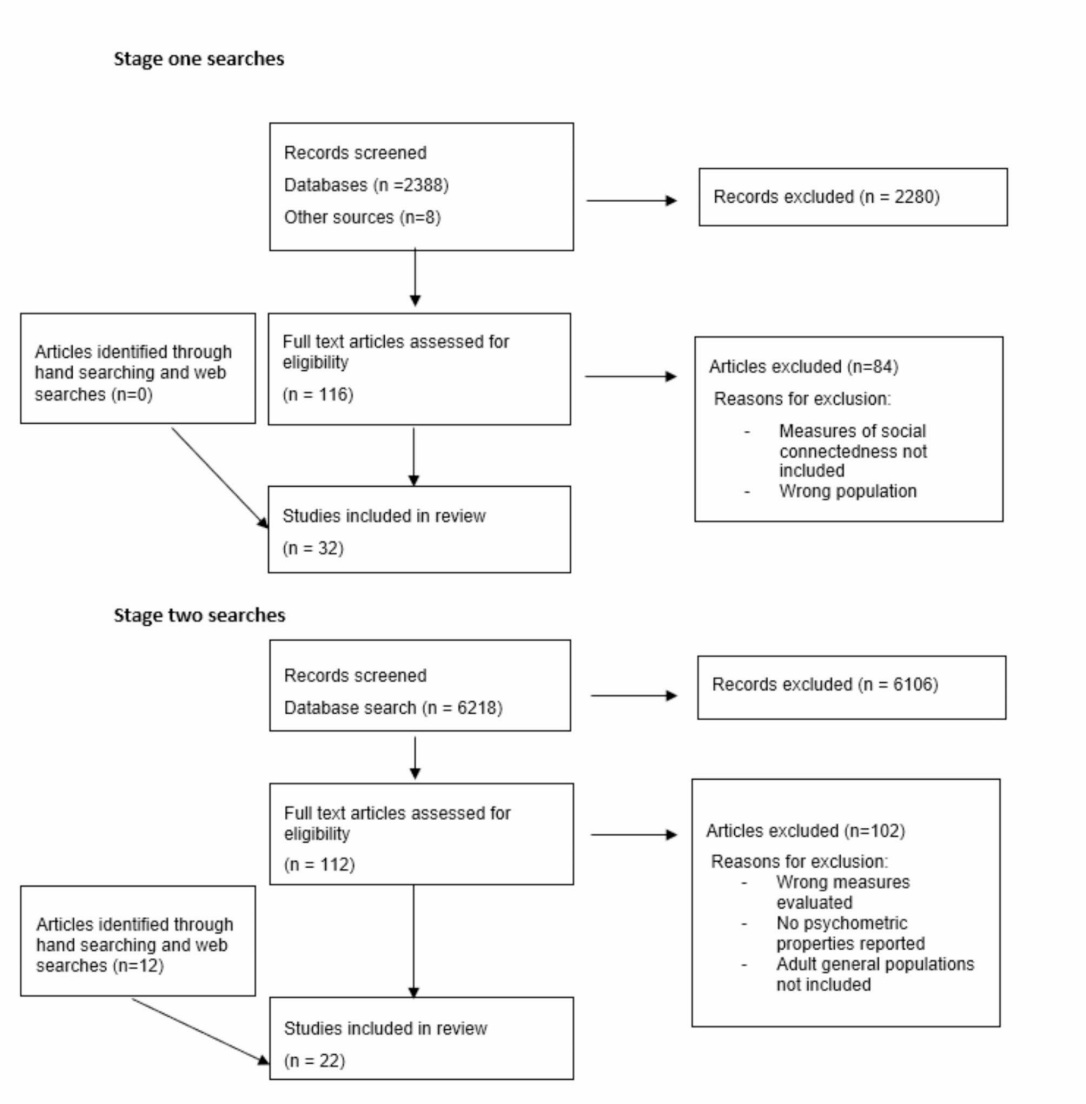
PRISMA flow diagram summarising the stage one and two social connectedness measures search process

After removing duplicates, our search yielded 2,396 papers for title and abstract screening. We screened the full text of 116 articles, and identified 32 review articles as suitable for inclusion. From the included review articles, we identified a total of 184 potential measures of social connectedness. Of these, 24 measures were deemed to potentially meet the operational definition of social connectedness and were taken forward to the second stage of searching. Figure 1 shows the PRISMA flowchart illustrating the selection process. Details of the included stage one studies are presented in Additional file 2.

#### Stage two

In searches for the evaluations of the psychometric properties of these measures, we identified 6,218 records. Of these 112 records were taken forward to full text screening and we deemed 10 of these records to be suitable for inclusion. We identified a further 12 through hand searching of reference lists and web searches. Therefore, we included 22 articles in our analysis and Fig. 1 shows the PRISMA diagram of the selection process. From these 22 articles, psychometric evidence was provided on 10 measures of social connectedness. These included four measures that were not identified in stage one searches (UCLA-7, SPS-5, SPS-10, F-SozU K-6). However, we did not undertake further citation searching of stage 2 results as the names of the measures were already addressed in the stage two search strategy. We excluded 18 of the 24 measures identified in stage one searches. Five of these were excluded due to additional information being gathered that indicated the included items did not meet our definition of social connectedness (e.g. items were focussed solely on frequency of contacts or relationships with healthcare professionals). A further three scales were removed because they were no longer made available by the developers, or we could not access the included items. Finally, 10 measures were excluded due to a lack of evidence of validation in relevant populations. These excluded measures are listed in Additional File 3. Most of the included measures assessed feelings of loneliness (*n* = 6) and four focussed on perceived social support; 40% (*n* = 4) were developed after the year 2000 and Table 2 provides further details of the measures.

**Table 2:** Characteristics of the included measures of social connectedness

Name	Author(s)	Year	Description	Cost/permissions to use	No. of items
**Included measures of loneliness**					
De Jong Gierveld Loneliness Scale (11-item)	De Jong Gierveld [24]	1985	A measure of emotional loneliness (missing intimate relationships), social loneliness (missing wider social networks) and overall loneliness.	Available if correct citations used and applied in survey research and not as a diagnostic test.	11
De Jong Gierveld Loneliness Scale (6 item)	De Jong Gierveld & Tilberg [25]	2006	Abbreviated measure of emotional loneliness (missing intimate relationships), social loneliness (missing wider social networks) and overall loneliness.	Available if correct citations used and applied in survey research and not as a diagnostic test.	6
Revised UCLA Loneliness scale (R-UCLA)	Russell [26]	1980	Revised version of the UCLA Loneliness scale that includes positively and negatively worded items to produce an overall measure of feelings of loneliness.	Not reported (NR)	20
UCLA Loneliness scale 7-item (UCLA-7)	Allen [27]	1995	Abbreviated version of the UCLA Loneliness scale, which retained seven items that all relate to friendship ties.	NR	7
UCLA Loneliness scale (version 3)	Russell [28]	1996	Simplified version of the R-UCLA. This scale attempts to address previous issues with the response format and wording of items included in the R-UCLA to provide an overall measure of perceived loneliness.	NR	20
Three-item Loneliness scale	Hughes [29]	2004	Short scale derived from R-UCLA. Provides measure of overall perceived loneliness.	NR	3
**Included measures of social support**					
Multidimensional Scale of Perceived Social Support (MSPSS)	Zimet et al. [30]	1988	A measure of perceived adequacy of available social support from friends, family, and significant others.	Free to use.	12
Perceived Social Support Questionnaire (F-SozU K-6)	Kliem et al. [31]	2015	A brief form measure of general perceived social support.	NR	6
Social Provisions Scale 10 (SPS-10)	Caron [32]	1996	Measure of perceived social support which assesses availability of social support, emotional support or attachment, social integration, reassurance of worth, tangible help and guidance.	NR	5
Social Provisions Scale 5 (SPS-5)	Orpana et al. [33]	2019	Shortened version of SPS 10 that provides a measure of overall perceived social support.	NR	5

Characteristics of the included studies from stage two are summarised in Tables 3 and 4. Table 3 describes journal article publications that reported on the reliability and validity of social connectedness measures in UK general adult populations (*n* = 10). Table 4 describes various types of publications, such as survey manuals and development papers, that reported on the pragmatic features of included social connectedness measures, such as scoring and respondent burden (*n* = 12). Just over half (55%, *n* = 12) of articles were published after the year 2000 and most studies were undertaken in the US (27%, *n* = 6). The most frequently reported measure was the R-UCLA (27%, *n* = 6), and the SPS-5 and UCLA-7 were least reported (4.6%, *n* = 1) respectively) [27, 33]. Sample sizes ranged from 58 − 22,486 [33, 34] and the average age across the six studies that reported age was 52. In almost all studies that reported gender, there were slightly more females than males (range: 49.5–65.6%). The most commonly reported mode of completion for the measure was interviewer administered on paper (18%, *n* = 4), and the least common reported modes were Computer Assisted Personal Interview and self-completion on paper (4.6%, *n* = 1 respectively) [29, 33]. The psychometric proprieties of each measure are described in Table 5.

**Table 3:** Characteristics of included studies from journal articles reporting on the reliability and validity of social connectedness measures

Author	Year	Measure(s)	Country	Sample size	Age			Gender (% female)	Mode of completion
					Mean	SD	Range		
Penning [35]	2014	De Jong Gierveld Loneliness Scale (11 item) & R-UCLA	Canada	Baseline = 243 Follow up = 204	Not reported (NR)	NR	45–84	54	Interviewer administered paper
Hyland [36]	2019	De Jong Gierveld Loneliness Scale (6 item)	US	1839	44.55	14.89	18–70	52	Self-complete online
Knight [37]	1988	R-UCLA	New Zealand	1120 (Complete data on 978)	NR	NR	16–89	49.49	NR
Cyranowski [38]	2013	R-UCLA	US	692	43.97	16.73	18+	56.6	NR
Allen [27]	1995	R- UCLA UCLA-7	US	619	NR	NR	18–87	60	Interview administered paper
Eglit [39]	2018	UCLA (version 3)	US	106	51.49	11.40	NR	55.66	NR
Hughes [29]	2004	Three-item Loneliness scale	US	229	57.5	4.4	50–67	52.4	Self-complete paper & Interviewer administered paper
Cartwright [34]	2020	MSPSS	UK	Overall = 270 Test-retest sample = 58	60.5	14.4	20–92	65.6	Self-complete (online)
Lin [40]	2018	F-SozU K-6	US	3038	55.12	17.50	18–99	58.8	Interviewer administered paper
Orpana [33]	2019	SPS-5 & SPS-10	Canada	Canadian Community Health Survey (CCHS) 2012 = 22,486 CCHS 2017 = 15,189	NR	NR	18+	CCHS 2012: 50.84 CCHS 2017: 50.27	Computer Assisted Personal Interview (CAPI)

**Table 4:** Characteristics of included studies from development papers or methodological guidance reporting on the pragmatic features of included social connectedness measures

Author	Year	Measure(s)	Country	Publication Type
Russell et al. [26]	1980	R-UCLA	US	Development paper
Russell et al. [41]	1978	R-UCLA	US	Development paper
Russell et al. [28]	1996	UCLA (version 3)	US	Development paper
Office for National Statistics [42]	2018	Three-item Loneliness Scale	UK	Methodological guidance
Zimet et al. [43]	1988	MSPSS	US	Development paper
Zimet [44]	Not reported (NR)	MSPSS	NR	Methodological guidance
De Jong Gierveld & Van Tilburg [45]	2021	De Jong Gierveld Loneliness scale (6-item)	Netherlands	User manual
De Jong Gierveld & Van Tilburg [25]	2006	De Jong Gierveld Loneliness scale (6-item)	Netherlands	Development paper
De Jong Gierveld & Kamphuis [24]	1985	De Jong Gierveld Loneliness scale (6-item)	Netherlands	Development paper
De Jong Gierveld [46]	1989	De Jong Gierveld Loneliness scale (11-item)	Netherlands	Development paper
Kliem et al. [31]	2015	F-SozU K-6	Germany	Development paper
Curtona et al. [47]	1983	SPS-10	US	Development paper

**Table 5:** Psychometric assessment of included measures

	Loneliness measures							Social support measures				
		De Jong Gierveld Loneliness Scale (11-item)	De Jong Gierveld Loneliness Scale (6 item)	Revised UCLA Loneliness scale (R-UCLA)	UCLA Loneliness scale 7-item (UCLA-7)	UCLA Loneliness scale version 3 (UCLA-3)	Three-item Loneliness scale		Multidimensional Scale of Perceived Social Support (MSPSS)	Perceived Social Support Questionnaire (F-SozU K-6)	Social Provisions Scale 10 (SPS-10)	Social Provisions Scale 5 (SPS-5)
CONCEPTUAL MODEL												
Construct defined?		✓	✓	✓	✓	✓	✓		✓	✓	✓	✓
Respondent population defined?		✘	✘	✘	✓	✓	✘		✘	✓	✓	✓
Conceptual model addresses whether a single or multiple subscales		✓	✓	✓	✓	✓	✘		✓	✓	✓	✓
CONTENT VALIDITY												
Respondent population involved in development?		✓	✘	✘	✘	✘	✘		✘	✘	✘	✘
Content experts involved in development?		✘	✘	✘	✘	✘	✘		✘	✘	✘	✘
Description of the methodology by which items/questions were determined?		✓	✘	✘	✘	✘	✘		✘	✘	✘	✘
RELIABILITY												
Reliability tested (e.g. test-retest, internal consistency? )		✘	✓	✓	✓	✓	✓		✓	✓	✓	✓
Reliability adequate (e.g. ideal: *r* ≥ .80; adequate 7 ≥ 0.70		✘	✓	✓	✓	✓	✓		✓	✓	✓	✓
CONSTRUCT VALIDITY												
Quantitative justification that single or multiple subscales exist (e.g. factor analysis or response theory)?		✓	✘	✘	✓	✓	✘		✓	✓	✘	✓
Associations with existing measures/ other relevant data		✘	✘	✓	✘	✓	✓		✓	✓	✓	✓
Expected differences in scores between relevant known groups		✘	✘	✘	✓	✓	✘		✘	✓	✘	✘
Measures change over time? both test-retest reliability AND responsiveness to change)?		✘	✘	✘	✘	✘	✘		✘	✘	✘	✘
SCORING AND INTERPRETATION												
Documentation on how to score measure?		✓	✓	✓	✘	✓	✓		✓	✓	✓	✓
How to score incomplete surveys described?		✓	✓	✘	✘	✘	✘		✘	✘	✓	✘
Information about interpreting scores?		✓	✓	✓	✘	✓	✓		✓	✓	✓	✓
RESPONDENT BURDEN AND PRESENTATION												
Time to complete reported and reasonable OR the number of questions appropriate for application?		✓	✓	✓	✓	✓	✓		✓	✓	✓	✓
Description of the literacy level?		✘	✘	✘	✘	✘	✘		✘	✘	✘	✘
Available for public viewing?		✓	✓	✓	✓	✓	✓		✓	✓	✓	✓

### Psychometric properties of identified measures

#### Loneliness measures

##### Conceptual model

The concept of loneliness was clearly defined for all the included scales. All three versions of the UCLA scales were designed to provide a global, unidimensional measure of loneliness focussing on the social domain of loneliness (R-UCLA, UCLA-3, UCLA-7) [26–28, 39]. The three-item loneliness scale also aims to provide a general measure of loneliness, but the structure of the measure was not clearly addressed within the included papers. Both De Jong Gierveld Loneliness scales clearly defined loneliness as multidimensional, encompassing both social and emotional domains of loneliness [24, 25, 45, 46]. Only two scales clearly defined their intended respondents, with both the UCLA-7 and UCLA-3 developed for use across a range of populations [27, 28].

##### Content validity

There was a paucity of evidence to support content validity, with only the 11-item De Jong Gierveld Loneliness scale meeting any of the included criteria. Here it was reported that the Dutch general population were involved in the original scale development. Content analysis and semi-structured face-to-face interviews were undertaken to determine the included items [24, 46]. There was evidence that staff members at the Department of Research Methods at the Free University of Amsterdam were also involved in evaluating items for inclusion in both this scale and the 6-item version [24]. However, it was unclear from the included articles whether these individuals could be considered content experts.

##### Reliability

Evidence of good internal consistency was reported for five of the included scales (R-UCLA, UCLA-7, UCLA-3, Three-item loneliness scale and De Jong Gierveld Loneliness scale (6-item) [27, 29, 36–39]. However, internal consistency coefficients appear only to have been reported when testing a two-factor model of the De Jong Gierveld Loneliness scale, in which one item was cross loaded on both factors [35].

##### Construct validity

Overall, three of the included measures reported a quantitative justification, such as factor analysis, for the proposed structure of their scale (De Jong Gierveld 11-item, UCLA-7, UCLA-3) [27, 35, 39]. Three scales also showed evidence for construct validity through significant associations with measures of related constructs (R-UCLA, UCLA-3, Three-item loneliness scale) [29, 38, 39]. However, evidence that the measure differentiates between groups known to differ on the variable of interest was weak across all measures. There is some limited evidence that both the UCLA-3 and UCLA-7 measures were associated with lower income and socioeconomic status [27, 39].

##### Scoring and interpretation

Documentation on scoring and how to interpret scores was available for all measures, aside from the UCLA-7 [25, 26, 28, 29, 36, 37, 42, 45]. Details on how to manage missing data was only identified for the 6-item and 11-item De Jong Gierveld Loneliness scales [45].

##### Respondent burden and presentation

All scales included 20 or fewer items and were deemed suitable for use in general populations. All scales were also available for public viewing; however, terms of use were only identified for the 6-item and 11-item De Jong Gierveld Loneliness scales [24, 25, 45]. Both of these scales are reported as being free to use if cited correctly and not used as a diagnostic test. The minimum level of literacy required to complete the scale was not reported for any of the included measures.

#### Social support measures

##### Conceptual model

Three of the included scales met all criteria relating to the conceptual model (F-SozU K-6, SPS-5, SPS-10), with the MSPSS meeting 2/3 criteria. The construct of social support was clearly defined for each of the measures, with two measures providing unidimensional measures of support (SPS-5, F-Sozu K-6) [33, 40] and one including five subscales relating to various social needs (SPS-10) [33]. The MSPSS was developed to provide an overall measure of support which also included subscales relating to support from family, friends and significant others [43]. Three of the included scales were developed for use in general populations [31, 33, 40], with the intended respondents of the MSPSS not clearly defined in the included literature.

##### Content validity

There was a paucity of evidence of content validity across the four included measures, with all measures failing to meet any of the criteria. There was some evidence that the original long form version of the F-SozU K-6 scale (F-SoZu) was evaluated in a community sample in Germany during the development process [31].

##### Reliability

Indices of internal consistency were reported for all scales, with good to excellent reliability demonstrated [33, 34, 40].

##### Construct validity

Justification for the intended structure of the measures were provided for the MSPSS, F-SoZu- K6 and the SPS-5 [33, 34, 40]. All scales showed evidence of construct validity through confirmed expected associations with other measures of mental health [33, 34, 40]. There was also some limited support that the F-SoZu K6 measure differentiates between groups known to differ on the variable of interest, through the finding that females demonstrated higher levels of social support as measured by the F-SoZu K6 as was hypothesised by the study authors [40]. None of the included studies fully satisfied the criteria relating to measurement change over time.

##### Scoring and interpretation

Guidance on scoring methods and scale interpretation were available for all included measures [31, 33, 40, 44]. However, documentation on how to manage and interpret missing data was only identified for the SPS-10 [33].

##### Respondent burden

The number of items included in each scale were deemed suitable for use in the general population and all scales were available for public viewing [31, 33, 40, 44]. However, details on permission of use were only available for the MSPSS, with it being reported that this scale was free to administer. The minimum level of literacy required to complete the scale was not reported for any of the included measures.

## Discussion

This review identified 24 measures of social connectedness for the general adult population and found psychometric evidence for 10 measures. The measures covered two key domains of the concept of social connectedness: subjective feelings of loneliness and perceived adequacy of social support. We found 22 studies which reported on the psychometric properties of 10 of the identified measures, but psychometric evidence was incomplete. Only five measures (50%) reported at least half of the psychometric criteria, and these were: UCLA-3 (for loneliness), and MSPSS, F-SozU K-6, SPS-10 and SPS-5 (for social support). A lack of reporting of the content validity of the measures was the most common methodological issue. Evidence on responsiveness to change and required literacy levels were lacking for all included scales. However, the included scales demonstrated evidence of good reliability overall.

This review highlights the inconsistencies in how aspects of social connectedness such as loneliness and social support are conceptualised and measured. Researchers and practitioners need to be aware of how these factors have been conceptualised when selecting measures, so that they can appropriately interpret and compare findings from these measures across studies. For example, the R-UCLA and De Jong Loneliness Gierveld scales are commonly used to measure loneliness, and this review also found those measures to have the most research related to their psychometric properties [5, 35, 48]. However, there are differences in the way these measures conceptualise loneliness. The R-UCLA measure focuses on the social domain, rather than the emotional domain of loneliness, and conceptualises loneliness as unidimensional [35, 36]. Whereas the De Jong Loneliness scale conceptualises loneliness as multidimensional and has items encompassing both domains of loneliness. Several empirical studies have found limited evidence to support the unidimensional conceptualisation of loneliness in the R-UCLA [27, 35, 39]. There is also some evidence that multidimensional measures are less likely to underrepresent the prevalence of loneliness in the population and may provide a more comprehensive measure to assess social connectedness at a population level [36].

The lack of a consensus and definition of social connectedness in general also makes it challenging to measure this concept and to interpret and compare findings in empirical studies that aim to establish risk factors, prevalence, or evaluate interventions to improve social connectedness [2]. This review focused on two domains that stakeholders prior to the review identified as most important for the concept of social connectedness, but other domains of social connectedness relating to trust, discrimination, safety, and sense of community were also identified as important by our stakeholders. Further work is required to identify reliable and valid measures addressing these domains. It is also likely that the concept of social connectedness varies across population groups and further research is needed to understand how the meaning of concepts like loneliness may vary across factors like age, gender, income status, culture and ethnicity [27]. Most studies in this review reported no content validity of the measures, so it is unclear whether the available measures fully capture all relevant aspects of social connectedness across adult populations. More research is needed to establish content validity, to understand how adults in the general population and experts perceive these measures and how accurately these measures capture the concept of social connectedness across groups.

### Strengths and limitations

This review was the first to identify and synthesise evidence on the psychometric properties of measures of social connectedness in the adult general population and encompassed measures of loneliness and social support. A rigorous, two-stage, review process was undertaken, which was informed by extensive stakeholder engagement to identify relevant domains of social connectedness. However, due to the range and scope of the literature, it was not feasible to identify measures relating to all the domains of social connectedness identified in the initial stakeholder workshops. The searches were conducted in 2020 but to the best of our knowledge no new measures or reviews have been published since these searches were conducted. This review was also limited as stage one searches relied on identifying measures through previous review articles. Therefore, there is potential that new measures, that have not yet been included in published systematic reviews, were not identified. We also only included studies in the English language and studies from English speaking countries with similar cultural values, which may have limited our findings, but this criterion was applied to make more informed recommendations for measures in a UK context. All the measures included in this study have been validated for non-English languages and future research is needed to synthesise the psychometric properties of these measures for use in other languages and countries.

There were also challenges in applying some of the included psychometric assessment, due to original items included in the criteria being developed for use in patient-reported measures rather than the general population. For example, the evidence on expected differences between groups was difficult to apply, as there is inconsistent evidence in previous literature relating to how certain groups are expected to vary in measures of social connectedness. The review was also limited by the quality of the studies, with many of the studies having relatively small sample sizes. Few studies explored whether loneliness and social support were conceptualised differently across demographic characteristics and by culture and whether scores differed based on these factors [27, 40].

It should also be noted that work has already been undertaken by the ONS on establishing a measure of loneliness suitable for use in the UK general population [10]. During this process further support for the validity and reliability of the three-item loneliness scale, has been reported in ONS guidance [10]. However, this report did not meet the strict review inclusion criteria, due to it not being published in a peer-reviewed journal. The current review aimed to go beyond the work conducted by ONS by identifying other aspects of social connectedness, such as social support and undertaking a thorough psychometric assessment of each measure.

## Conclusions

This review evaluated a dense and complex body of literature covering various domains of social connectedness. Ten measures of social connectedness were identified with an evaluation of their psychometric properties, which are potentially suitable for use in the UK adult general population. The social connectedness measures with the most evidence for their psychometric properties were UCLA-3, MSPSS, F-SozUK-6, SPS-10, and SPS-5. The identification and psychometric assessment of these social connectedness measures reported in this review could contribute towards the development of a core outcome set for measuring social connectedness at population level. More research is needed to clarify the definition and conceptualisation of social connectedness to ensure this concept is meaningfully captured and to establish content validity of current measures. This could enable standardisation in the approach to measuring social connectedness, which will help researchers, practitioners, and policymakers to make more informed decisions about the effectiveness of public mental health interventions.

## Electronic supplementary material

Below is the link to the electronic supplementary material.

Supplementary Material 1

Supplementary Material 2

Supplementary Material 3

Supplementary Material 4

## Data Availability

Not applicable.

## Abbreviations

CAPI: Computer Assisted Personal Interview
CCHS: Canadian Community Health Survey
F-SozUK-6: Fragebogen zur Sozialen Unterstützung Kurzform mit sechs Items
MSPSS: The Multidimensional Scale of Perceived Social Support
NR: Not reported
ONS: Office of National Statistics
PRISMA: Preferred Reporting Items for Systematic Reviews and Meta-Analyses
R-UCLA: Revised University of California, Los Angeles loneliness Scale
SPHR: School for Public Health Research
SPS-10 and SPS-5: Social Provisions Scale 10 and 5
UCLA-3: The University of California, Los Angeles version 3
UCLA-7: The University of California, Los Angeles ten-item loneliness scale
