# Supplementary material for: Measures of social connectedness in adult populations: a systematic review
Source: BMC Public Health. 2024 Dec 5;24:3384. doi: 10.1186/s12889-024-20779-0 (PMC11622465; doi:10.1186/s12889-024-20779-0)
Supplement: Supplementary file 1 — Supplementary Material 1 [file 12889_2024_20779_MOESM1_ESM.docx]

**Additional File 1 – Search transcripts for all databases for stages 1 and 2**

**MEDLINE**

**Stage 1**

| 1. | Resilience, Psychological/ or self-concept/ or self-efficacy/ or Hope/ or Happiness/ or Personal satisfaction/ or Optimism/ or Personal autonomy/ |
| --- | --- |
| 2. | (wellbeing or well being or resilience or selfconfidence or self confidence or happiness or happy or life satisfaction or satisfied with life or eudemonia or eudemonic or eudaimonic or eudaimonia or hedonism or hedonic or cheerful* or hope or optimism).mp. |
| 3. | 1 or 2 |
| 4. | (public mental health or population mental health or public health or population health or health promotion or psychiatric epidemiology or preventive psychiatry).mp. |
| 5. | Public Health/ or Preventive psychiatry/ or Epidemiologic Studies/ or Health promotion/ |
| 6. | 4 or 5 |
| 7. | "Surveys and Questionnaires"/ |
| 8. | (measurement or objective measure* or subjective measure* or evaluative measure* or tool or instrument or scale or inventor* or indicator* or survey or question* or questionnaire* or wemwbs or "Warwick and edinburgh mental wellbeing scale" or what works wellbeing or european social survey or annual population survey or ONS4 or office for national statistics or UK household longitudinal study or cantril scale or euro barometer life satisfaction or eurobarometer life satisfaction or perma model or day reconstruction method or affectometer 2).mp. |
| 9. | 7 or 8 |
| 10. | health status indicators/ |
| 11. | "outcome and process assessment (health care)"/ |
| 12. | "outcome assessment (health care)"/ |
| 13. | quality of life/ |
| 14. | health status/ |
| 15. | severity of illness index/ |
| 16. | self assessment/ |
| 17. | outcome measure$.tw. |
| 18. | health outcome$.tw. |
| 19. | quality of life.tw. |
| 20. | health status.tw. |
| 21. | (endpoint$ or end point$ or end-point$).tw. |
| 22. | (self-report$ or self report$).tw. |
| 23. | functional outcome$.tw. |
| 24. | outcome$.ti. |
| 25. | or/10-24 |
| 26. | outcome$.tw. |
| 27. | measure$.tw. |
| 28. | assess$.tw. |
| 29. | (score$ or scoring).tw. |
| 30. | index.tw. |
| 31. | indices.tw. |
| 32. | scale$.tw. |
| 33. | monitor$.tw. |
| 34. | or/27-33 |
| 35. | 26 and 34 |
| 36. | 25 or 35 |
| 37. | 9 or 36 |
| 38. | 3 and 6 and 37 |
| 39. | meta analysis.mp,pt. or review.pt. or search:.tw. |
| 40. | 38 and 39 |

**Stage 2**

1 UCLA loneliness.mp.

2 ("UCLA scale" and (lone* or alone or isolat* or social*)).mp.

3 (R-UCLA or RUCLA).mp.

4 loneliness index.mp.

5 (russell and peplau).mp.

6 (short scale and lonel*).mp.

7 de Jong Gierveld.mp.

8 (SELSA or SELSA-S).mp.

9 emotional loneliness scale.mp.

10 friendship scale.mp.

11 perceived social support scale.mp.

12 PSS scale.mp.

13 (PSS-FR or PSS-FA).mp.

14 procidano.mp.

15 personal resource questionnaire.mp.

16 weinert.mp.

17 (brandt and social).mp.

18 Multidimensional Scale of Perceived Social Support.mp.

19 Multi-dimensional Scale of Perceived Social Support.mp.

20 MSPSS.mp.

21 (zimet or canty-mitchell).mp.

22 social support questionnaire.mp.

23 (SSQ3 or SSQ 3).mp.

24 (SSQ6 or SSQ 6).mp.

25 sarason.mp.

26 (medical outcome study and social support survey).mp.

27 (MOS and social support survey).mp.

28 MOS-SS.mp.

29 Duke-UNC.mp.

30 ("functional social support" adj3 (survey or questionnaire)).mp.

31 FSSQ.mp.

32 broadhead.mp.

33 social provisions scale.mp.

34 cutrona.mp.

35 ("sense of belonging" adj2 (instrument or scale or measure)).mp.

36 Interpersonal Support Evaluation List.mp.

37 ISEL.mp.

38 campaign to end loneliness.mp.

39 (instrumentation or methods).fs. or Validation Studies.pt. or Comparative Study.pt. or exp psychometrics/ or psychometr*.ti,ab. or clinimetr*.mp. or clinometr*.mp. or exp "Outcome Assessment (Health Care)"/ or outcome assessment.ti,ab. or outcome measure*.mp. or exp observer variation/ or observer variation.ti,ab. or exp Health Status Indicators/ or exp reproducibility of results/ or reproducib*.ti,ab. or exp discriminant analysis/ or reliab*.ti,ab. or unreliab*.ti,ab. or valid*.ti,ab. or coefficient.ti,ab. or homogeneity.ti,ab. or homogeneous.ti,ab. or internal consistency.ti,ab. or (cronbach* and (alpha or alphas)).ti,ab. or (item and (correlation* or selection* or reduction*)).ti,ab. or agreement.ti,ab. or precision.ti,ab. or imprecision.ti,ab. or precise values.ti,ab. or test-retest.ti,ab. or (test and retest).ti,ab. or (reliab* and (test or retest)).ti,ab. or stability.ti,ab. or interrater.ti,ab. or inter-rater.ti,ab. or intrarater.ti,ab. or intra-rater.ti,ab. or intertester.ti,ab. or inter-tester.ti,ab. or intratester.ti,ab. or intra-tester.ti,ab. or interobserver.ti,ab. or inter-observer.ti,ab. or intraobserver.ti,ab. or intra-observer.ti,ab. or intertechnician.ti,ab. or inter-technician.ti,ab. or intratechnician.ti,ab. or intra-technician.ti,ab. or interexaminer.ti,ab. or inter-examiner.ti,ab. or intraexaminer.ti,ab. or intra-examiner.ti,ab. or interassay.ti,ab. or inter-assay.ti,ab. or intraassay.ti,ab. or intra-assay.ti,ab. or interindividual.ti,ab. or inter-individual.ti,ab. or intraindividual.ti,ab. or intra-individual.ti,ab. or interparticipant.ti,ab. or inter-participant.ti,ab. or intraparticipant.ti,ab. or intra-participant.ti,ab. or kappa.ti,ab. or kappa's.ti,ab. or kappas.ti,ab. or repeatab*.ti,ab. or ((replicab* or repeated) and (measure or measures or findings or result or results or test or tests)).ti,ab. or generaliza*.ti,ab. or generalisa*.ti,ab. or concordance.ti,ab. or (intraclass and correlation*).ti,ab. or discriminative.ti,ab. or known group.ti,ab. or factor analysis.ti,ab. or factor analyses.ti,ab. or dimension*.ti,ab. or subscale*.ti,ab. or (multitrait and scaling and (analysis or analyses)).ti,ab. or item discriminant.ti,ab. or interscale correlation*.ti,ab. or error.ti,ab. or errors.ti,ab. or individual variability.ti,ab. or (variability and (analysis or values)).ti,ab. or (uncertainty and (measurement or measuring)).ti,ab. or standard error of measurement.ti,ab. or sensitiv*.ti,ab. or responsive*.ti,ab. or ((minimal or minimally or clinical or clinically) and (important or significant or detectable) and (change or difference)).ti,ab. or (small* and (real or detectable) and (change or difference)).ti,ab. or meaningful change.ti,ab. or ceiling effect.ti,ab. or floor effect.ti,ab. or Item response model.ti,ab. or IRT.ti,ab. or Rasch.ti,ab. or Differential item functioning.ti,ab. or DIF.ti,ab. or computer adaptive testing.ti,ab. or item bank.ti,ab. or cross-cultural equivalence.ti,ab.

40 instrumentation.fs. or Validation Studies.pt. or exp reproducibility of results/ or reproducib*.ti,ab. or exp psychometrics/ or psychometr*.ti,ab. or clinimetr*.ti,ab. or clinometr*.ti,ab. or exp observer variation/ or observer variation.ti,ab. or exp discriminant analysis/ or reliab*.ti,ab. or valid*.ti,ab. or coefficient.ti,ab. or internal consistency.ti,ab. or (cronbach* and (alpha or alphas)).ti,ab. or item correlation.ti,ab. or item correlations.ti,ab. or item selection.ti,ab. or item selections.ti,ab. or item reduction.ti,ab. or item reductions.ti,ab. or agreement.mp. or precision.mp. or imprecision.mp. or precise values.mp. or test-retest.ti,ab. or (test and retest).ti,ab. or (reliab* and (test or retest)).ti,ab. or stability.ti,ab. or interrater.ti,ab. or inter-rater.ti,ab. or intrarater.ti,ab. or intra-rater.ti,ab. or intertester.ti,ab. or inter-tester.ti,ab. or intratester.ti,ab. or intra-tester.ti,ab. or interobserver.ti,ab. or inter-observer.ti,ab. or intraobserver.ti,ab. or intra-observer.ti,ab. or intertechnician.ti,ab. or inter-technician.ti,ab. or intratechnician.ti,ab. or intra-technician.ti,ab. or interexaminer.ti,ab. or inter-examiner.ti,ab. or intraexaminer.ti,ab. or intra-examiner.ti,ab. or interassay.ti,ab. or inter-assay.ti,ab. or intraassay.ti,ab. or intra-assay.ti,ab. or interindividual.ti,ab. or inter-individual.ti,ab. or intraindividual.ti,ab. or intra-individual.ti,ab. or interparticipant.ti,ab. or inter-participant.ti,ab. or intraparticipant.ti,ab. or intra-participant.ti,ab. or kappa.ti,ab. or kappa*.ti,ab. or coefficient of variation.ti,ab. or repeatab*.mp. or ((replicab* or repeated) and (measure or measures or findings or result or results or test or tests)).mp. or generaliza*.ti,ab. or generalisa*.ti,ab. or concordance.ti,ab. or (intraclass and correlation*).ti,ab. or discriminative.ti,ab. or known group.ti,ab. or factor analysis.ti,ab. or factor analyses.ti,ab. or factor structure.ti,ab. or factor structures.ti,ab. or dimensionality.ti,ab. or subscale*.ti,ab. or multitrait scaling analysis.ti,ab. or multitrait scaling analyses.ti,ab. or "item discriminantOR interscale correlation".ti,ab. or interscale correlations.ti,ab. or ((error or errors) and (measure* or correlat* or evaluat* or accuracy or accurate or precision or mean)).ti,ab. or individual variability.ti,ab. or interval variability.ti,ab. or rate variability.ti,ab. or variability analysis.ti,ab. or (uncertainty and (measurement or measuring)).ti,ab. or standard error of measurement.ti,ab. or sensitiv*.ti,ab. or responsive*.ti,ab. or (limit and detection).ti,ab. or minimal detectable concentration.ti,ab. or interpretab*.ti,ab. or (small* and (real or detectable) and (change or difference)).ti,ab. or meaningful change.ti,ab. or minimal important change.ti,ab. or minimal important difference.ti,ab. or minimally important change.ti,ab. or minimally important difference.ti,ab. or minimal detectable change.ti,ab. or minimal detectable difference.ti,ab. or minimally detectable change.ti,ab. or minimally detectable difference.ti,ab. or minimal real change.ti,ab. or minimal real difference.ti,ab. or minimally real change.ti,ab. or minimally real difference.ti,ab. or ceiling effect.ti,ab. or floor effect.ti,ab. or Item response model.ti,ab. or IRT.ti,ab. or Rasch.ti,ab. or Differential item functioning.ti,ab. or DIF.ti,ab. or computer adaptive testing.ti,ab. or item bank.ti,ab. or cross-cultural equivalence.ti,ab.

41 or/1-38

42 (39 or 40) and 41

43 40 and 41

44 exp Neoplasms/

45 exp Pregnancy/

46 exp Patients/

47 44 or 45 or 46

48 43 not 47

**EMBASE**

**Stage 1:**

| 1. | psychological resilience/ or self concept/ or hope/ or happiness/ or satisfaction/ or optimism/ or personal autonomy/ |
| --- | --- |
| 2. | (wellbeing or well being or resilience or selfconfidence or self confidence or happiness or happy or life satisfaction or satisfied with life or eudemonia or eudemonic or eudaimonic or eudaimonia or hedonism or hedonic or cheerful* or hope or optimism).mp. |
| 3. | 1 or 2 |
| 4. | (public mental health or population mental health or public health or population health or health promotion or psychiatric epidemiology or preventive psychiatry).mp. |
| 5. | public Health/ or social psychiatry/ or epidemiology/ or health promotion/ |
| 6. | 4 or 5 |
| 7. | questionnaire/ |
| 8. | (measurement or objective measure* or subjective measure* or evaluative measure* or tool or instrument or scale or inventor* or indicator* or survey or question* or questionnaire* or wemwbs or "Warwick and edinburgh mental wellbeing scale" or what works wellbeing or european social survey or annual population survey or ONS4 or office for national statistics or UK household longitudinal study or cantril scale or euro barometer life satisfaction or eurobarometer life satisfaction or perma model or day reconstruction method or affectometer 2).mp. |
| 9. | 7 or 8 |
| 10. | health status indicator/ |
| 11. | outcome assessment/ |
| 12. | "quality of life"/ |
| 13. | health status/ |
| 14. | "severity of illness index"/ |
| 15. | self evaluation/ |
| 16. | outcome measure$.tw. |
| 17. | health outcome$.tw. |
| 18. | quality of life.tw. |
| 19. | health status.tw. |
| 20. | (endpoint$ or end point$ or end-point$).tw. |
| 21. | (self-report$ or self report$).tw. |
| 22. | functional outcome$.tw. |
| 23. | outcome$.ti. |
| 24. | or/10-23 |
| 25. | outcome$.tw. |
| 26. | measure$.tw. |
| 27. | assess$.tw. |
| 28. | (score$ or scoring).tw. |
| 29. | index.tw. |
| 30. | indices.tw. |
| 31. | scale$.tw. |
| 32. | monitor$.tw. |
| 33. | or/26-32 |
| 34. | 25 and 33 |
| 35. | 24 or 34 |
| 36. | 9 or 35 |
| 37. | 3 and 6 and 36 |
| 38. | meta-analys:.mp. or search:.tw. or review.pt. |
| 39. | 37 and 38 |

**Stage 2:**

1 instrumentation.sh.

2 methods.sh.

3 Validation Stud*.mp.

4 Comparative Stud*.mp.

5 Psychometry/

6 psychometr*.ti,ab.

7 clinimetr*.tw.

8 clinometr*.tw.

9 Outcome Assessment/

10 outcome assessment.ti,ab.

11 outcome measure*.tw.

12 Observer Variation/

13 observer variation.ti,ab.

14 Health Status Indicator/

15 Reproducibility/

16 reproducib*.ti,ab.

17 Discriminant Analysis/

18 reliab*.ti,ab.

19 unreliab*.ti,ab.

20 valid*.ti,ab.

21 coefficient.ti,ab.

22 homogeneity.ti,ab.

23 homogeneous.ti,ab.

24 "internal consistency".ti,ab.

25 1 or 2 or 3 or 4 or 5 or 6 or 7 or 8 or 9 or 10 or 11 or 12 or 13 or 14 or 15 or 16 or 17 or 18 or 19 or 20 or 21 or 22 or 23 or 24

26 cronbach*.ti,ab.

27 (alpha or alphas).ti,ab.

28 26 and 27

29 item.ti,ab.

30 (correlation* or selection* or reduction*).ti,ab.

31 29 and 30

32 (agreement or precision or imprecision or "precise values" or "test–retest").ti,ab.

33 (test and retest).ti,ab.

34 reliab*.ti,ab.

35 (test or retest).ti,ab.

36 34 and 35

37 28 or 31 or 32 or 33 or 36

38 (stability or interrater or inter-rater or intrarater or intra-rater or intertester or inter-tester or intratester or intra-tester or interobserver or inter-observer or intraobserver or intra-observer).ti,ab.

39 (intertechnician or inter-technician or intratechnician or intra-technician or interexaminer or inter-examiner or intraexaminer or intra-examiner or interassay or inter-assay or intraassay or intra-assay or interindividual or inter-individual or intraindividual or intra-individual or interparticipant or inter-participant or intraparticipant or intra-participant).ti,ab.

40 (kappa or "kappa’s" or kappas or repeatab*).ti,ab.

41 repeatab*.ti,ab.

42 38 or 39 or 40 or 41

43 (replicab* or repeated).ti,ab.

44 (measure or measures or findings or result or results or test or tests).ti,ab.

45 43 and 44

46 (generaliza* or generalisa* or concordance).ti,ab.

47 (intraclass and correlation*).ti,ab.

48 (discriminative or "known group" or factor analysis or factor analyses or dimension* or subscale*).ti,ab.

49 (multitrait and scaling and (analysis or analyses)).ti,ab.

50 45 or 46 or 47 or 48 or 49

51 (item discriminant or interscale correlation* or error or errors or "individual variability").ti,ab.

52 (variability and (analysis or values)).ti,ab.

53 (uncertainty and (measurement or measuring)).ti,ab.

54 ("standard error of measurement" or sensitiv* or responsive*).ti,ab.

55 ((minimal or minimally or clinical or clinically) and (important or significant or detectable) and (change or difference)).ti,ab.

56 (small* and (real or detectable) and (change or difference)).ti,ab.

57 (meaningful change or "ceiling effect" or "floor effect" or "Item response model" or IRT or Rasch or "Differential item functioning" or DIF or "computer adaptive testing" or "item bank" or "cross-cultural equivalence").ti,ab.

58 51 or 52 or 53 or 54 or 55 or 56 or 57

59 25 or 37 or 42 or 50 or 58

60 UCLA loneliness.mp.

61 ("UCLA scale" and (lone* or alone or isolat* or social*)).mp.

62 (R-UCLA or RUCLA).mp.

63 loneliness index.mp.

64 (russell and peplau).mp.

65 (short scale and lonel*).mp.

66 de Jong Gierveld.mp.

67 (SELSA or SELSA-S).mp.

68 emotional loneliness scale.mp.

69 friendship scale.mp.

70 perceived social support scale.mp.

71 PSS scale.mp.

72 (PSS-FR or PSS-FA).mp.

73 procidano.mp.

74 personal resource questionnaire.mp.

75 weinert.mp.

76 (brandt and social).mp.

77 Multidimensional Scale of Perceived Social Support.mp.

78 Multi-dimensional Scale of Perceived Social Support.mp.

79 MSPSS.mp.

80 (zimet or canty-mitchell).mp.

81 social support questionnaire.mp.

82 (SSQ3 or SSQ 3).mp.

83 (SSQ6 or SSQ 6).mp.

84 sarason.mp.

85 (medical outcome study and social support survey).mp.

86 (MOS and social support survey).mp.

87 MOS-SS.mp.

88 Duke-UNC.mp.

89 (("functional social support" or (FSS and (social* or lonel*))) adj3 (survey or questionnaire)).mp.

90 FSSQ.mp.

91 broadhead.mp.

92 social provisions scale.mp.

93 cutrona.mp.

94 ("sense of belonging" adj2 (instrument or scale or measure)).mp.

95 Interpersonal Support Evaluation List.mp.

96 campaign to end loneliness.mp.

97 or/60-96

98 59 and 97

99 remove duplicates from 98

100 limit 99 to conference abstracts

101 99 not 100

102 limit 101 to english language

103 instrumentation.fs. or Validation Studies.pt. or exp reproducibility of results/ or reproducib*.ti,ab. or exp psychometrics/ or psychometr*.ti,ab. or clinimetr*.ti,ab. or clinometr*.ti,ab. or exp observer variation/ or observer variation.ti,ab. or exp discriminant analysis/ or reliab*.ti,ab. or valid*.ti,ab. or coefficient.ti,ab. or internal consistency.ti,ab. or (cronbach* and (alpha or alphas)).ti,ab. or item correlation.ti,ab. or item correlations.ti,ab. or item selection.ti,ab. or item selections.ti,ab. or item reduction.ti,ab. or item reductions.ti,ab. or agreement.mp. or precision.mp. or imprecision.mp. or precise values.mp. or test-retest.ti,ab. or (test and retest).ti,ab. or (reliab* and (test or retest)).ti,ab. or stability.ti,ab. or interrater.ti,ab. or inter-rater.ti,ab. or intrarater.ti,ab. or intra-rater.ti,ab. or intertester.ti,ab. or inter-tester.ti,ab. or intratester.ti,ab. or intra-tester.ti,ab. or interobserver.ti,ab. or inter-observer.ti,ab. or intraobserver.ti,ab. or intra-observer.ti,ab. or intertechnician.ti,ab. or inter-technician.ti,ab. or intratechnician.ti,ab. or intra-technician.ti,ab. or interexaminer.ti,ab. or inter-examiner.ti,ab. or intraexaminer.ti,ab. or intra-examiner.ti,ab. or interassay.ti,ab. or inter-assay.ti,ab. or intraassay.ti,ab. or intra-assay.ti,ab. or interindividual.ti,ab. or inter-individual.ti,ab. or intraindividual.ti,ab. or intra-individual.ti,ab. or interparticipant.ti,ab. or inter-participant.ti,ab. or intraparticipant.ti,ab. or intra-participant.ti,ab. or kappa.ti,ab. or kappa*.ti,ab. or coefficient of variation.ti,ab. or repeatab*.mp. or ((replicab* or repeated) and (measure or measures or findings or result or results or test or tests)).mp. or generaliza*.ti,ab. or generalisa*.ti,ab. or concordance.ti,ab. or (intraclass and correlation*).ti,ab. or discriminative.ti,ab. or known group.ti,ab. or factor analysis.ti,ab. or factor analyses.ti,ab. or factor structure.ti,ab. or factor structures.ti,ab. or dimensionality.ti,ab. or subscale*.ti,ab. or multitrait scaling analysis.ti,ab. or multitrait scaling analyses.ti,ab. or (item discriminant or interscale correlation).ti,ab. or interscale correlations.ti,ab. or ((error or errors) and (measure* or correlat* or evaluat* or accuracy or accurate or precision or mean)).ti,ab. or individual variability.ti,ab. or interval variability.ti,ab. or rate variability.ti,ab. or variability analysis.ti,ab. or (uncertainty and (measurement or measuring)).ti,ab. or standard error of measurement.ti,ab. or sensitiv*.ti,ab. or responsive*.ti,ab. or (limit and detection).ti,ab. or minimal detectable concentration.ti,ab. or interpretab*.ti,ab. or (small* and (real or detectable) and (change or difference)).ti,ab. or meaningful change.ti,ab. or minimal important change.ti,ab. or minimal important difference.ti,ab. or minimally important change.ti,ab. or minimally important difference.ti,ab. or minimal detectable change.ti,ab. or minimal detectable difference.ti,ab. or minimally detectable change.ti,ab. or minimally detectable difference.ti,ab. or minimal real change.ti,ab. or minimal real difference.ti,ab. or minimally real change.ti,ab. or minimally real difference.ti,ab. or ceiling effect.ti,ab. or floor effect.ti,ab. or Item response model.ti,ab. or IRT.ti,ab. or Rasch.ti,ab. or Differential item functioning.ti,ab. or DIF.ti,ab. or computer adaptive testing.ti,ab. or item bank.ti,ab. or cross-cultural equivalence.ti,ab.

104 102 and 103

105 exp neoplasm/

106 104 not 105

**APA PsycInfo**

**Stage 1:**

| 1. | "Resilience (Psychological)"/ or Self-Concept/ or Self-Efficacy/ or Hope/ or Happiness/ or Satisfaction/ or Optimism/ or Autonomy/ or Life Satisfaction/ or exp Well being/ |
| --- | --- |
| 2. | (wellbeing or well being or resilience or selfconfidence or self confidence or happiness or happy or life satisfaction or satisfied with life or eudemonia or eudemonic or eudaimonic or eudaimonia or hedonism or hedonic or cheerful* or hope or optimism).mp. |
| 3. | 1 or 2 |
| 4. | (public mental health or population mental health or public health or population health or health promotion or psychiatric epidemiology or preventive psychiatry).mp. |
| 5. | Public Health/ or Epidemiology/ or Health Promotion/ |
| 6. | 4 or 5 |
| 7. | exp Surveys/ or Questionnaires/ |
| 8. | (measurement or objective measure* or subjective measure* or evaluative measure* or tool or instrument or scale or inventor* or indicator* or survey or question* or questionnaire* or wemwbs or "Warwick and edinburgh mental wellbeing scale" or what works wellbeing or european social survey or annual population survey or ONS4 or office for national statistics or UK household longitudinal study or cantril scale or euro barometer life satisfaction or eurobarometer life satisfaction or perma model or day reconstruction method or affectometer 2).mp. |
| 9. | 7 or 8 |
| 10. | Health status/ |
| 11. | "Treatment Process and Outcome Measures"/ |
| 12. | "Quality of Life"/ |
| 13. | Self-Evaluation/ |
| 14. | outcome measure$.tw. |
| 15. | health outcome$.tw. |
| 16. | quality of life.tw. |
| 17. | health status.tw. |
| 18. | (endpoint$ or end point$ or end-point$).tw. |
| 19. | (self-report$ or self report$).tw. |
| 20. | functional outcome$.tw. |
| 21. | outcome$.ti. |
| 22. | or/10-21 |
| 23. | outcome$.tw. |
| 24. | measure$.tw. |
| 25. | assess$.tw. |
| 26. | (score$ or scoring).tw. |
| 27. | index.tw. |
| 28. | indices.tw. |
| 29. | scale$.tw. |
| 30. | monitor$.tw. |
| 31. | or/24-30 |
| 32. | 23 and 31 |
| 33. | 22 or 32 |
| 34. | 9 or 33 |
| 35. | 3 and 6 and 34 |
| 36. | (control: or effectiveness or risk:).tw. |
| 37. | 35 and 36 |

**Stage 2:**

| 1. | UCLA loneliness scale.mp. |
| --- | --- |
| 2. | ("UCLA scale" and (lone* or alone or isolat* or social*)).mp. |
| 3. | (R-UCLA or RUCLA).mp. |
| 4. | loneliness index.mp. |
| 5. | (russell and peplau).mp. |
| 6. | (short scale and lonel*).mp. |
| 7. | de Jong Gierveld.mp. |
| 8. | (SELSA or SELSA-S).mp. |
| 9. | emotional loneliness scale.mp. |
| 10. | friendship scale.mp. |
| 11. | perceived social support scale.mp. |
| 12. | PSS scale.mp. |
| 13. | (PSS-FR or PSS-FA).mp. |
| 14. | procidano.mp. |
| 15. | personal resource questionnaire.mp. |
| 16. | weinert.mp. |
| 17. | (brandt and social).mp. |
| 18. | Multidimensional Scale of Perceived Social Support.mp. |
| 19. | Multi-dimensional Scale of Perceived Social Support.mp. |
| 20. | MSPSS.mp. |
| 21. | (zimet or canty-mitchell).mp. |
| 22. | social support questionnaire.mp. |
| 23. | (SSQ3 or SSQ 3).mp. |
| 24. | (SSQ6 or SSQ 6).mp. |
| 25. | sarason.mp. |
| 26. | (medical outcome study and social support survey).mp. |
| 27. | (MOS and social support survey).mp. |
| 28. | MOS-SS.mp. |
| 29. | Duke-UNC.mp. |
| 30. | (("functional social support" or (FSS and (social* or lonel*))) adj3 (survey or questionnaire)).mp. |
| 31. | FSSQ.mp. |
| 32. | broadhead.mp. |
| 33. | social provisions scale.mp. |
| 34. | cutrona.mp. |
| 35. | ("sense of belonging" adj2 (instrument or scale or measure)).mp. |
| 36. | Interpersonal Support Evaluation List.mp. |
| 37. | campaign to end loneliness.mp. |
| 38. | or/1-37 |
| 39. | (instrumentation or Validation Stud* or reproducib* or psychometr* or clinimetr* or clinometr* or observer variation or discriminant analysis or reliab* or valid* or coefficient or internal consistency or (cronbach* and (alpha or alphas)) or item correlation or item correlations or item selection or item selections or item reduction or item reductions or agreement or precision or imprecision or precise values or (test and retest) or (reliab* and (test or retest)) or stability or interrater or inter rater or intrarater or intra rater or intertester or inter tester or intratester or intra-tester or interobserver or inter observer or intraobserver or intra observer or intertechnician or inter technician or intratechnician or intra technician or interexaminer or inter examiner or intraexaminer or intra examiner or interassay or inter assay or intraassay or intra assay or interindividual or inter individual or intraindividual or intra individual or interparticipant or inter participant or intraparticipant or intra participant or kappa or kappas or coefficient of variation or repeatab* or ((replicab* or repeated) and (measure or measures or findings or result or results or test or tests)) or generaliza* or generalisa* or concordance or (intraclass and correlation*) or discriminative or known group or factor analysis or factor analyses or factor structure or factor structures or dimensionality or subscale* or multitrait scaling analysis or multitrait scaling analyses or item discriminant or interscale correlation or interscale correlations or ((error or errors) and (measure* or correlat* or evaluat* or accuracy or accurate or precision or mean)) or individual variability or interval variability or rate variability or variability analysis or (uncertainty and (measurement or measuring)) or standard error of measurement or sensitiv* or responsive* or (limit and detection) or minimal detectable concentration or interpretab* or (small* and (real or detectable) and (change or difference)) or meaningful change or minimal important change or minimal important difference or minimally important change or minimally important difference or minimal detectable change or minimal detectable difference or minimally detectable change or minimally detectable difference or minimal real change or minimal real difference or minimally real change or minimally real difference or ceiling effect or floor effect or Item response model or IRT or Rasch or Differential item functioning or DIF or computer adaptive testing or item bank or cross cultural equivalence).mp. |
| 40. | 38 and 39 |
| 41. | limit 40 to english language |
| 42. | exp neoplasms/ or exp physical disorders/ |
| 43. | 41 not 42 |

| **CINAHL - STAGE 1:** |
| --- |

| **#** | **Query** |
| --- | --- |
| S12 | S10 AND S11 |
| S11 | ( review or review of literature or literature review or meta-analysis or systematic review ) OR ( synthesis or overview ) |
| S10 | S8 AND S9 |
| S9 | ( measurement or "objective measure*" or "subjective measure*" or "evaluative measure*" or tool or instrument or scale or inventor* or indicator* or survey or question* or questionnaire* or wemwbs or "Warwick and edinburgh mental wellbeing scale" or "european social survey" or "annual population survey" or ONS4 or "office for national statistics" or "UK household longitudinal study" or "cantril scale" or "perma model" or "day reconstruction method" or affectometer 2 ) OR ( scor* or questionnaire ) |
| S8 | S1 AND S4 |
| S7 | S1 AND S4 |
| S6 | S1 AND S4 |
| S5 | S1 AND S4 |
| S4 | S2 OR S3 |
| S3 | (fear or perception* or anxiet* or concerns or concerned) n3 (crime* or criminal or "antisocial behavio*" or "anti-social behavio*" or danger or violence or safety) ) OR ( (trust* or mistrust or distrust or cohesion) n3 (communit* or neighbo*) ) OR ( (bullying or intimidat* or cyberbully*) ) OR "community spirit" |
| S2 | ( "social* connect*" or "social* isolat*" or "social* disconnect*" or lonel* or disenfranchis* or "social network*" or "support network*" or "sense of community" or (community n2 participat*) ) OR ( "social capital" or "social cohesion" or "societal cohesi*" or interconnect* or interdepen* or reciproc* or "social integrat*" ) OR ( community resilience or community resiliency ) OR ( sense of community or sense of belonging ) OR ( discriminat* or stigma* ) OR ( (toleran* or acceptan* or integrat* or inclu*) n3 (racial* or cultural* or sectarian* or ethnic* or divers* or disab* or handicap* or sexuality or LGBT* or minorit* or marginali*) ) |
| S1 | ( "public mental health" or "population mental health" or "public health" or "population health" or "health promotion" or "psychiatric epidemiology" or "preventive psychiatry" ) OR ( "public Health" or "social psychiatry" or epidemiology or "health promotion" ) |

**CINAHL STAGE 2:**

|  |
| --- |

| **#** | **Query** |
| --- | --- |
| S16 | S12 OR S15 |
| S15 | S13 AND S14 |
| S14 | ( reproducib* or methods or valid* or "reproducibility of results" or reliab* ) OR ( "internal consistency" or "ceiling effect" or coefficient or "co-efficient" or "observer variation" ) OR ( psychometric* or discriminative or precision ) |
| S13 | "personal resource questionnaire" OR ("PRQ-82" or "PRQ-85" or "PRQ-2000") |
| S12 | S1 OR S2 OR S3 OR S5 OR S6 OR S7 OR S8 OR S9 OR S10 OR S11 |
| S11 | "campaign to end loneliness" |
| S10 | "interpersonal support evaluation list" or ISEL |
| S9 | "sense of belonging instrument" |
| S8 | "social provisions scale" |
| S7 | Duke-UNC OR "functional social support questionnaire" OR FSSQ OR ( FSS and "social support" ) |
| S6 | "social support questionnaire" OR ( SSQ or "SSQ 3" or "SSQ 6" or SSQ3 or SSQ6 or SSQ-3 or SSQ-6 ) |
| S5 | "Multidimensional Scale of Perceived Social Support" OR MSPSS OR "Multi-dimensional Scale of Perceived Social Support" |
| S4 | "personal resource questionnaire" OR ("PRQ-82" or "PRQ-85" or "PRQ-2000") |
| S3 | "friendship scale" OR "perceived social support scale" OR ( "PSS scale" or PSS-FR or PSS-FA ) OR "perceived social support" n4 "scale" |
| S2 | de Jong Gierveld OR ( "social and emotional loneliness scale" ) OR ( SELSA or SELSA-S ) |
| S1 | UCLA Loneliness OR ( RUCLA or R-UCLA ) |

**Web of Science – all indexes**

Search history: <https://www.webofscience.com/wos/woscc/summary/5c0ef6d2-262f-40e7-8d68-b41c575957d8-011a34f631/relevance/1>

## Websites included in stage 1 searches

| **Website/organisation** | **URL** |
| --- | --- |
| Resilience Research Centre | https://resilienceresearch.org/ |
| What Works Centre for Wellbeing | https://whatworkswellbeing.org/ |
| Mind | https://www.mind.org.uk/ |
| American Psychological Association | https://www.apa.org/ |
| Al Siebert Resiliency Center | https://resiliencycenter.com/ |
| American Addiction Centres | https://www.mentalhelp.net/ |
| PositivePsychology.com | https://positivepsychology.com/ |
| Mental Health Foundation | https://mentalhealthfoundation.org/ |
| The McPin Foundation | https://mcpin.org/ |
| The Centre for Mental Health | https://www.centreformentalhealth.org.uk/ |
| The Loneliness and Social Isolation in Mental Health Network (UCL) | https://www.ucl.ac.uk/psychiatry/research/epidemiology-and-applied-clinical-research-department/loneliness-and-social-isolation |
| Campaign to End Loneliness | https://www.campaigntoendloneliness.org/ |
| Linking Lives UK | Linkinglives.uk |
| Samuel Center for Social Connectedness | https://www.socialconnectedness.org/about-us/ |
