# Supplementary material for: Measures of social connectedness in adult populations: a systematic review
Source: BMC Public Health. 2024 Dec 5;24:3384. doi: 10.1186/s12889-024-20779-0 (PMC11622465; doi:10.1186/s12889-024-20779-0)
Supplement: Supplementary file 2 — Supplementary Material 2 [file 12889_2024_20779_MOESM2_ESM.docx]

## Additional file 2

## Characteristics of studies included in stage 1 social connectedness search

| Author | Year | Design | Aim | Studies included (N) |
| --- | --- | --- | --- | --- |
| Britton [1] | 2020 | Systematic review | Identify and evaluate the impact of blue space interventions | 33 |
| Clement [2] | 2013 | Systematic review | Assess the effects of mass media interventions on mental health-related stigma | 22 |
| Cohen-Mansfield [3] | 2015 | Critical review | Evaluate the effectiveness of loneliness interventions in older adults | 34 |
| Deckx [4] | 2018 | Systematic literature review | To assess the relationship between loneliness and coping strategies | 12 |
| Dickens [5] | 2011 | Systematic review | Assess the effectiveness of loneliness and social isolation interventions in older adults | 32 |
| Douglas [6] | 2017 | Narrative review | Review the association between social participation and health in older adults | NR |
| Dyal [7] | 2015 | Systematic review | Assess the association between smoking and loneliness | 25 |
| Escobar-Viera [8] | 2018 | Systematic review | Assess the relationship between social media use and depression in lesbian, gay and bisexual individuals. | 11 |
| Hallisy [9] | 2018 | Review of reviews (narrative?) | Provide a historical perspective on Thai Chi and associations with health and social isolation. | NR |
| Hunter [10] | 2019 | Meta-narrative evidence synthesis | To assess the impact of urban green space on health, wellbeing, social, environmental and equity. | 38 |
| Husk [11] | 2016 | Systematic review | Assess the impact of participation in nature conservation and related activities on health. | 19 |
| Jenkinson [12] | 2013 | Systematic review and meta-analysis | Examine the impact of volunteering on physical and mental health and survival. | 40 |
| Leigh-hunt [13] | 2017 | Systematic review of systematic reviews | Assess the wider impact of loneliness and isolation on public health. | 40 |
| Mancus [14] | 2018 | Systematic review/integrative review | Evaluate the relationship between green space and neighbourhood violence. | 30 |
| Nicholson [15] | 2012 | Literature review | Review literature on the impact of isolation in older adults. | 70 |
| Noel [16] | 2018 | Systematic review | Examine the conceptualisation and measurement of social capital and relationships with other variables in post-disaster/conflict zones | 15 |
| Poscia [17] | 2018 | Systematic review | Evaluate the effectiveness of interventions for loneliness and isolation in older adults. | 20 |
| Rees-Punia [18] | 2018 | Meta-analysis | Examine the relationship between crime, perceived safety and physical activity | 16 |
| Samari [19] | 2018 | Systematic review | Assess associations between islamophobia and public health | 53 |
| Smith [20] | 2017 | Systematic review | Evaluate the association between social support and physical activity in older adults | 27 |
| Valtorta [21] | 2018 | Systematic review | Determine the association between older adults’ social relationships and health care utilisation. | 126 |
| Veazie [22] |  | Rapid review | Evaluate the effectiveness of interventions which aim to alleviate loneliness and social isolation on health and/or health care utilisation. | 16 |
| Villalonga-Olives [23] | 2015 | Systematic review | Examine bridging social capital in public health settings | 23 |
| Villalonga-Olives [24] | 2017 | Systematic review | Evaluate the negative associations between social capital and health | 44 |
| Villalonga-Olives [25] | 2018 | Systematic review | Evaluate the effectiveness of social capital interventions in public health | 17 |
| Wang [26] | 2017 | Conceptual and methodological review | Develop a framework for social isolation and related concepts and identify relevant measures | 702 |
| Xue [27] | 2020 | Meta-analysis | Evaluate the relationships between social capital and health | 470 |
| Victor  (What Works Centre for Wellbeing) [28] | 2018 | Review of reviews | To determine the effectiveness of interventions to alleviate loneliness | 14 |
| Scherer (What Works Centre for Wellbeing) [29] | 2019 | Blog/guide | Provide guidance on measuring loneliness | NA |
| What Works Centre for Wellbeing [30] | 2020 | Report | Assess the impact of COVID-19 on loneliness in the UK | NA |
| The Campaign to End Loneliness [31] | NR | Report | Provide guidance on choosing scales to measure the impact of services on loneliness in older adults | NA |
| Griffiths [32] | NR | Report | Provide an overview of loneliness and social isolation in the UK | NA |

References

1. Britton E, Kindermann G, Domegan C, Carlin C: **Blue care: a systematic review of blue space interventions for health and wellbeing**. *Health Promotion International* 2020, **35**(1):50-69.

2. Clement S, Lassman F, Barley E, Evans-Lacko S, Williams P, Yamaguchi S, Slade M, Rüsch N, Thornicroft G: **Mass media interventions for reducing mental health-related stigma**. *Cochrane Database Syst Rev* 2013(7):Cd009453.

3. Cohen-Mansfield J, Perach R: **Interventions for alleviating loneliness among older persons: a critical review**. *Am J Health Promot* 2015, **29**(3):e109-125.

4. Deckx L, van den Akker M, Buntinx F, van Driel M: **A systematic literature review on the association between loneliness and coping strategies**. *Psychol Health Med* 2018, **23**(8):899-916.

5. Dickens AP, Richards SH, Greaves CJ, Campbell JL: **Interventions targeting social isolation in older people: a systematic review**. *BMC Public Health* 2011, **11**(1):647.

6. Douglas H, Georgiou A, Westbrook J: **Social participation as an indicator of successful aging: an overview of concepts and their associations with health**. *Aust Health Rev* 2017, **41**(4):455-462.

7. Dyal SR, Valente TW: **A Systematic Review of Loneliness and Smoking: Small Effects, Big Implications**. *Subst Use Misuse* 2015, **50**(13):1697-1716.

8. Escobar-Viera CG, Whitfield DL, Wessel CB, Shensa A, Sidani JE, Brown AL, Chandler CJ, Hoffman BL, Marshal MP, Primack BA: **For Better or for Worse? A Systematic Review of the Evidence on Social Media Use and Depression Among Lesbian, Gay, and Bisexual Minorities**. *JMIR Ment Health* 2018, **5**(3):e10496.

9. Hallisy KM: **Tai Chi Beyond Balance and Fall Prevention: Health Benefits and Its Potential Role in Combatting Social Isolation in the Aging Population**. *Current Geriatrics Reports* 2018, **7**(1):37-48.

10. Hunter RF, Cleland C, Cleary A, Droomers M, Wheeler BW, Sinnett D, Nieuwenhuijsen MJ, Braubach M: **Environmental, health, wellbeing, social and equity effects of urban green space interventions: A meta-narrative evidence synthesis**. *Environment International* 2019, **130**:104923.

11. Husk K, Lovell R, Cooper C, Stahl-Timmins W, Garside R: **Participation in environmental enhancement and conservation activities for health and well-being in adults: a review of quantitative and qualitative evidence**. *Cochrane Database Syst Rev* 2016, **2016**(5):CD010351-CD010351.

12. Jenkinson CE, Dickens AP, Jones K, Thompson-Coon J, Taylor RS, Rogers M, Bambra CL, Lang I, Richards SH: **Is volunteering a public health intervention? A systematic review and meta-analysis of the health and survival of volunteers**. *BMC Public Health* 2013, **13**(1):773.

13. Leigh-Hunt N, Bagguley D, Bash K, Turner V, Turnbull S, Valtorta N, Caan W: **An overview of systematic reviews on the public health consequences of social isolation and loneliness**. *Public Health* 2017, **152**:157-171.

14. Mancus GC, Campbell J: **Integrative Review of the Intersection of Green Space and Neighborhood Violence**. *J Nurs Scholarsh* 2018, **50**(2):117-125.

15. Nicholson NR: **A review of social isolation: an important but underassessed condition in older adults**. *J Prim Prev* 2012, **33**(2-3):137-152.

16. Noel P, Cork C, White RG: **Social Capital and Mental Health in Post-Disaster/Conflict Contexts: A Systematic Review**. *Disaster Med Public Health Prep* 2018, **12**(6):791-802.

17. Poscia A, Stojanovic J, La Milia DI, Duplaga M, Grysztar M, Moscato U, Onder G, Collamati A, Ricciardi W, Magnavita N: **Interventions targeting loneliness and social isolation among the older people: An update systematic review**. *Exp Gerontol* 2018, **102**:133-144.

18. Rees-Punia E, Hathaway ED, Gay JL: **Crime, perceived safety, and physical activity: A meta-analysis**. *Prev Med* 2018, **111**:307-313.

19. Samari G, Alcalá HE, Sharif MZ: **Islamophobia, Health, and Public Health: A Systematic Literature Review**. *Am J Public Health* 2018, **108**(6):e1-e9.

20. Lindsay Smith G, Banting L, Eime R, O’Sullivan G, van Uffelen JGZ: **The association between social support and physical activity in older adults: a systematic review**. *International Journal of Behavioral Nutrition and Physical Activity* 2017, **14**(1):56.

21. Valtorta NK, Moore DC, Barron L, Stow D, Hanratty B: **Older Adults' Social Relationships and Health Care Utilization: A Systematic Review**. *Am J Public Health* 2018, **108**(4):e1-e10.

22. Veazie S, Gilbert J, Winchell K, Paynter R, Guise JM: **AHRQ Rapid Evidence Product Reports**. In: *Addressing Social Isolation To Improve the Health of Older Adults: A Rapid Review.* edn. Rockville (MD): Agency for Healthcare Research and Quality (US); 2019.

23. Villalonga-Olives E, Kawachi I: **The measurement of bridging social capital in population health research**. *Health & Place* 2015, **36**:47-56.

24. Villalonga-Olives E, Kawachi I: **The dark side of social capital: A systematic review of the negative health effects of social capital**. *Soc Sci Med* 2017, **194**:105-127.

25. Villalonga-Olives E, Wind TR, Kawachi I: **Social capital interventions in public health: A systematic review**. *Social Science & Medicine* 2018, **212**:203-218.

26. Wang J, Lloyd-Evans B, Giacco D, Forsyth R, Nebo C, Mann F, Johnson S: **Social isolation in mental health: a conceptual and methodological review**. *Soc Psychiatry Psychiatr Epidemiol* 2017, **52**(12):1451-1461.

27. Xue X, Reed WR, Menclova A: **Social capital and health: a meta-analysis**. *Journal of Health Economics* 2020, **72**:102317.

28. Victor C, Mansfield L, Kay T, Daykin N, Lane J, Grigsby Duffy L, Tomlinson A, Meads C: **An overview of reviews: the effectiveness of interventions to address loneliness at all stages of the life-course**. In*.*; 2018.

29. Scherer IA: **Measuring loneliness: new guidance**. In*.*: What Works Centre for Wellbeing; 2019.

30. What Works Centre for Wellbeing (2020). **"How has Covid-19 and associated lockdown measures affected loneliness in the UK?"**. Retrieved 9th September, 2024, from <https://whatworkswellbeing.org/resources/loneliness-lockdown-and-covid/>.

31. Campaign to End Loneliness (2018). **"Measuring your Impact on Loneliness in Later Life."** Retrieved 9th September, 2024, from <https://www.campaigntoendloneliness.org/wp-content/uploads/Loneliness-Measurement-Guidance1.pdf>.

32. Griffiths H (2017). **"Social Isolation and Loneliness in the UK."** Retrieved 28th October, 2024, from <https://cp-catapult.s3.amazonaws.com/uploads/2021/06/Social-Isolation-and-Loneliness-Landscape-UK.pdf>.
