# Supplementary material for: Measures of social connectedness in adult populations: a systematic review
Source: BMC Public Health. 2024 Dec 5;24:3384. doi: 10.1186/s12889-024-20779-0 (PMC11622465; doi:10.1186/s12889-024-20779-0)
Supplement: Supplementary file 3 — Supplementary Material 3 [file 12889_2024_20779_MOESM3_ESM.docx]

| Name | Author(s) | Year | Description |
| --- | --- | --- | --- |
| UCLA Loneliness Scale | Russel et al.[1] | 1978 | Original version of the UCLA Loneliness scale includng 20 negatively worded items. |
| Social and Emotional Loneliness Scale for Adults (SELSA) | DiTomasso & Spinner[2] | 1993 | Multidimensional measure of loneliness which distinguishes between social and emotional loneliness (romantic and family loneliness). |
| Short version of the social and emotional loneliness scale for adults (SELSA-S) | DiTomasso et al.[3] | 2004 | 15-item version of the SELSA, selected from the original romantic, family and social subscales. |
| The Friendship Scale | Hawthorne & Griffin[4] | 2000 | 6-item, user friendly scale developed to assess 6 dimensions of social connection. |
| Personal Resource Questionnaire 2000 (PRQ-2000) | Weinert[5] | 2000 | 15 positively worded questions used to assess perceived social support. Excludes the section on interpersonal resources which is assessed in previous PRQ scales. |
| Social Support Questionnaire (SSQ) | Sarason et al.[6] | 1983 | 27-item scale which assess perceptions and satisfaction with available social support. |
| Social Support Questionnaire 3 (SSQ 3) | Sarason et al.[7] | 1987 | 3-item version of SSQ. |
| Social Support Questionnaire 6 (SSQ 6) | Sarason et al.[7] | 1987 | 6-item version of SSQ. |
| Campaign to End Loneliness tool | Campaign to End Loneliness[8] | 2014 | 3-item measure of loneliness consisting of positively worded items. |
| Sense of Belonging instrument | Hagerty & Patusky[9] | 1995 | 27-item measure which consists of two scales: SOBI-P (psychological state) and SOBI-A (antecedents). |

Additional file 3

**Scales excluded due to lack of evidence on psychometric properties**

References

1. Russell D, Peplau LA, Ferguson ML: **Developing a Measure of Loneliness**. *Journal of Personality Assessment* 1978, **42**(3):290-294.

2. DiTommaso E, Spinner B: **The development and initial validation of the Social and Emotional Loneliness Scale for Adults (SELSA)**. *Personality and Individual Differences* 1993, **14**(1):127-134.

3. DiTommaso E, Brannen C, Best LA: **Measurement and Validity Characteristics of the Short Version of the Social and Emotional Loneliness Scale for Adults**. *Educational and Psychological Measurement* 2004, **64**(1):99-119.

4. Hawthorne G, Griffith P: **The friendship scale: Development and properties**: Centre for Health Program Evaluation Melbourne; 2000.

5. Weinert C: **Measuring social support: PRQ2000**. In: *Measurement of nursing outcomes: Vol 3 Self care and coping.* edn. Edited by Strickland O, DiIorio C. New York: Springer: 161-172.

6. Sarason IG, Levine HM, Basham RB, Sarason BR: **Assessing social support: The Social Support Questionnaire**. *Journal of Personality and Social Psychology* 1983, **44**:127-139.

7. Sarason IG, Sarason BR, Shearin EN, Pierce GR: **A Brief Measure of Social Support: Practical and Theoretical Implications**. *Journal of Social and Personal Relationships* 1987, **4**(4):497-510.

8. Knight RG, Chisholm BJ, Marsh NV, Godfrey HPD: **Some normative, reliability, and factor analytic data for the revised UCLA Loneliness scale**. *Journal of Clinical Psychology* 1988, **44**(2):203-206.

9. Hagerty BM, Patusky K: **Developing a measure of sense of belonging**. *Nurs Res* 1995, **44**(1):9-13.
